# Femtosecond laser micromachining of an optofluidics-based monolithic whispering-gallery mode resonator coupled to a suspended waveguide

**DOI:** 10.1038/s41598-021-88682-x

**Published:** 2021-04-28

**Authors:** João M. Maia, Vítor A. Amorim, Duarte Viveiros, P. V. S. Marques

**Affiliations:** 1 CAP – Centre for Applied Photonics, INESC TEC, 4150-179 Porto, Portugal; 2 Departament of Physics and Astronomy, Faculty of Sciences, University of Porto, 4169-007 Porto, Portugal

**Keywords:** Optofluidics, Microresonators, Laser material processing

## Abstract

A monolithic lab-on-a-chip fabricated by femtosecond laser micromachining capable of label-free biosensing is reported. The device is entirely made of fused silica, and consists of a microdisk resonator integrated inside a microfluidic channel. Whispering gallery modes are excited by the evanescent field of a circular suspended waveguide, also incorporated within the channel. Thermal annealing is performed to decrease the surface roughness of the microstructures to a nanometric scale, thereby reducing intrinsic losses and maximizing the Q-factor. Further, thermally-induced morphing is used to position, with submicrometric precision, the suspended waveguide tangent to the microresonator to enhance the spatial overlap between the evanescent field of both optical modes. With this fabrication method and geometry, the alignment between the waveguide and the resonator is robust and guaranteed at all instances. A maximum sensitivity of 121.5 nm/RIU was obtained at a refractive index of 1.363, whereas near the refractive index range of water-based solutions the sensitivity is 40 nm/RIU. A high Q-factor of 10^5^ is kept throughout the entire measurement range.

## Introduction

Optical microresonators support light propagation along its surface in the form of whispering-gallery modes (WGM), due to total internal reflection. The unique features of strong light confinement within a small volume and for long periods of time, promote light-matter interactions that make these devices ideal platforms for optical sensing^1,2^. Accordingly, the detection of several physical parameters, such as temperature^3^, pressure^4^, electric^5^ or magnetic^6^ field has already been demonstrated. Further, the progress made in signal enhancement techniques has contributed to rapid advances in the field. Detection through mode splitting^7^ or mode broadening^8^ enable self-reference measurements with a limit of detection at the nanometric scale, whereas plasmonic enhancement^9^, through integration of WGM with surface plasmon resonances, has led to an improvement in the sensitivity. Meanwhile, chemical functionalization of the surface of the microresonator has enabled targeted detection of single nanoparticles, molecules, or viruses^7,10^. Moreover, the construction of WGM resonators on materials with optical gain has enabled the production of low-threshold microlasers^11^.

Different geometries (e.g. microspheres^12^, microdisks^13^, or microbottles^14^) have also been exploited, with the WGMs being typically excited and collected through the near evanescent field of a free-standing tapered fiber^11^, through prism coupling^15^ or through a microstructured fiber containing a microsphere resonator in one of the holes at the tip of the fiber^16^. Despite presenting high sensitivities with ultrahigh quality (Q) factors^15^, the coupling technique itself limits the widespread use of these devices. The lack of integration between the microresonator and the coupling prism or tapered fiber impacts the portability and handling of the device, while the mechanical instability of the latter can also become a noise source and disturb the excitation of WGMs. Also, the incompatibility between traditional processing techniques prevents the integration of these optical devices with microfluidic systems capable of handling low volumes of analyte, which is appealing for biosensing and in medical environments.

Recently, femtosecond laser micromachining^17,18^ has been proposed as a versatile fabrication tool for material processing and device fabrication. Depending on the material and irradiation conditions, several effects can be observed, leading to a wide range of applications. Local increase of the refractive index led to the formation of optical waveguides and more complex optical systems^19^ in both passive and active media, whereas ablation^20^ or enhancement of etching selectivity^21^ enabled the construction of microfluidic systems in several glasses^18^. Meanwhile, multiphoton polymerization has been used to produce polymeric microstructures inside a microfluidic channel^22^ or at the tip of an optical fiber^23^. In fact, within the context of this work, two-photon polymerization proved to be successful in the fabrication of microresonators permanently coupled to a tapered waveguide, which is responsible for the excitation of WGMs^24,25^. However, the use of polymers can compromise the performance of this device, especially when compared to its silica-based counterparts. Fused silica, besides being the material of choice in optical communications, is also attractive for biosensing and biomedical applications due to its biocompatibility and inertness to solvents. Further, its mechanical durability and thermal stability is a pre-requisite in most real-life applications. Nevertheless, the fabrication of this geometry in fused silica has proven to be a challenge, owing to the subtractive nature of fs-laser irradiation followed by chemical etching^26^. Here, we report how the introduction of a subsequent thermal annealing procedure enables the production of a monolithic chip, entirely made of fused silica, which includes a WGM resonator within the microfluidic channel, for fluid handling capabilities, and a suspended waveguide responsible for permanently coupling light into the resonator. After describing the fabrication method, the optical response of the device against the refractive index of the fluid surrounding the microresonator is discussed.

## Results

### Description of the device

**Figure 1:**
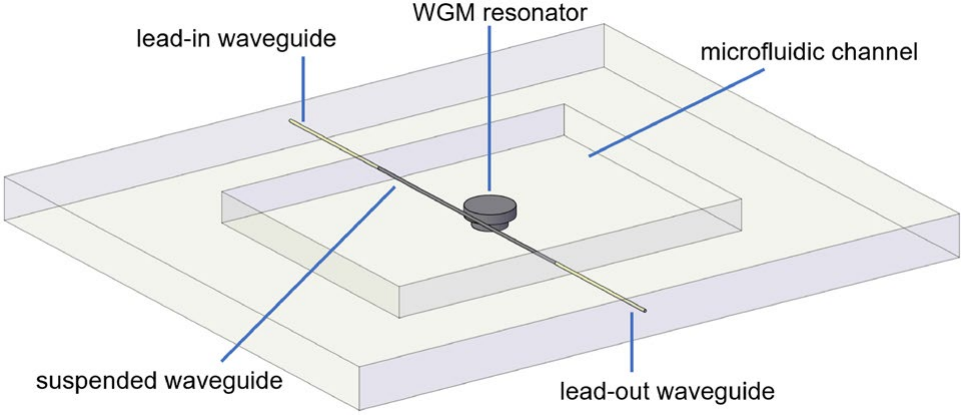
Schematic of the device. The microfluidic channel, containing the WGM resonator and the suspended waveguide, is first fabricated by fs-laser direct writing followed by chemical etching and thermal annealing. Afterwards, the lead-in and lead-out waveguides, axially aligned to the suspended waveguide, are fabricated through fs-laser direct writing.

A schematic of the device is shown in Fig. 1. It is composed by a microfluidic channel that contains a microdisk, supported by a cylindrical pedestal of lower radius, and a suspended waveguide, which is anchored to the lateral walls of the microfluidic channel. The suspended waveguide is tangent to the microdisk, and can thereby excite WGMs of the resonator through evanescent coupling. Two fs-laser written waveguides, denominated lead-in and lead-out waveguide, couple light into and out of the suspended waveguide, respectively. Fluids injected inside the microfluidic channel can directly interact with the optical resonator and with the suspended waveguide. The process flow for the fabrication of the monolithic sensor consists of three main steps, which are described in the Materials and Methods section: (1) fs-laser irradiation followed by chemical etching of the irradiated areas to create the microfluidic channel (already including the microresonator and the suspended waveguide), (2) thermal annealing of the substrate, and (3) fs-laser direct writing of the lead-in and lead-out waveguides.

The inclusion of thermal annealing to the fabrication process solves some of the limiting issues related to the fabrication and performance of the device. First, the surface roughness is inversely proportional to the intrinsic Q-factor of the microresonator^27^, and negatively affects the propagation losses of the suspended waveguide due to optical scattering. This parameter should then be minimized, which can be achieved through thermal treatment, either by thermal annealing^28^ or CO_2_ laser polishing^29^. Second, and more crucial, it allows to circumvent the limitations imposed by the chemical etching, and which are characteristic of fs-laser micromachining. In particular, the etching selectivity created by fs-laser writing is limited^21^ and, consequently, a hollow gap between the initially tangent microstructures is always formed, which determines that the device is in an under-coupling regime. In our system, a minimum gap of 2.5 µm is always observed, which prevents the efficient excitation of WGMs. To overcome this issue, a morphing technique, which is induced by heating the substrate to a temperature above its annealing point, was employed. Although the morphing effect has also been used to transform the shape of microstructures^29^, here, it can be utilized as a mechanism to place the suspended waveguide within the correct distance to achieve the critical coupling condition.

### Surface roughness

**Figure 2:**
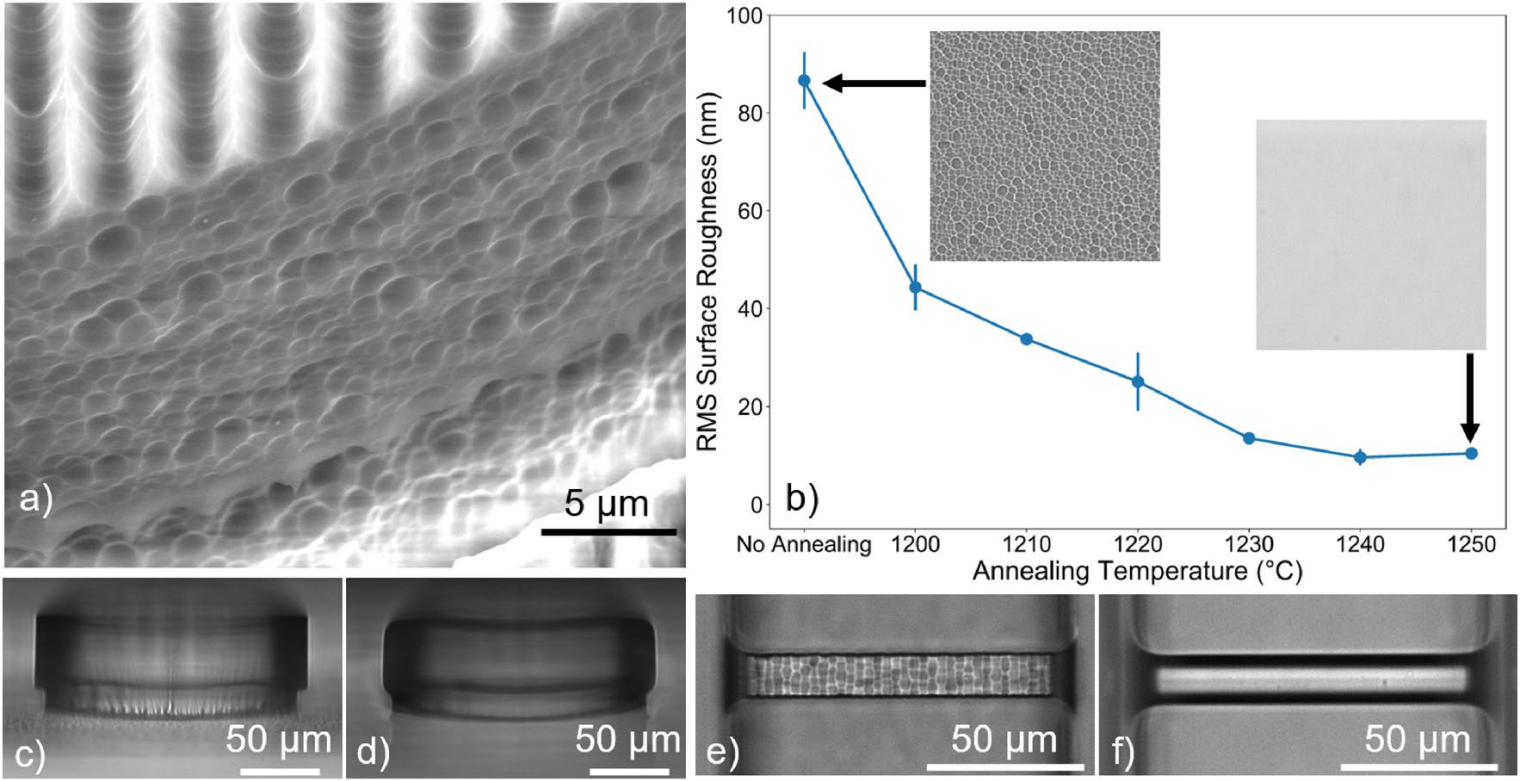
Surface roughness of the microstructures. (**a**) SEM image of a microdisk with radius and height of 75 µm by 50 µm after chemical etching. (**b**) Evolution of the RMS roughness with chemical etching and annealing temperature. The insets show the profile of the microfluidic channel’s surface after chemical etching and after thermal annealing at 1250 °C. (**c**, **d**) Cross-section image of the same microresonator before and after thermal annealing at 1250 °C, respectively. (**e**, **f**) Top view image of a 100-µm long suspended waveguide before and after thermal annealing at 1250 °C.

Despite obtaining a direct replica of the designed device, the chemical etching leads to an undesired surface profile. Figure 2a reveals that both the top and sidewall surfaces of the microdisk are rugged, albeit with different topographies. The top surface exhibits a periodical modulation along the direction transverse to the scanning orientation of the fs-laser beam, where the crests are spaced by 3 µm which matches the horizontal gap between adjacent laser scans. Meanwhile, the sidewall pattern is more random and smoother, which is associated to a lower surface roughness. This difference is related to the dimensions of a singular modification track and the user-defined spacing between each. During laser direct writing, the laser-irradiated tracks merge along the vertical direction which results in a more uniform etching, whereas they are separated by a thin pristine layer along the horizontal direction which creates a rougher profile after chemical etching. Although the effect observed in the top (and bottom surfaces) could be theoretically minimized by reducing the separation between horizontally-adjacent laser scans to below the defined 3 µm, in practice it was observed that this resulted in fractures in the material.

Therefore, in order to decrease the surface roughness, the substrate is subjected to a thermal treatment process. Figure 2b shows that the root-mean squared (RMS) surface roughness decreases asymptotically with the annealing temperature, which is also confirmed from the inset images of Fig. 2b and from Fig. 2e, f where it is shown that the initially rugged surface becomes smoother. The smoothing is due to material redistribution in an energetically favorable configuration as a result of the surface tension overcoming thermally decreasing viscosity forces^30^. Further, the material’s viscosity decreases as the temperature increases, which enhances the reflow effect and minimizes the surface roughness, thereby explaining the behavior against annealing temperature depicted in Fig. 2b. In particular, after thermal annealing at 1250 °C, the RMS roughness decreases from 87 to 11 nm, while the surface remains uniform which also indicates a homogeneous refractive index distribution. Moreover, for lengths up to 2000 µm, the suspended waveguide is straight and stable after thermal annealing, as demonstrated in Fig. 2f. For lengths greater than 2000 µm, the suspended waveguide started to bend downwards, as also highlighted by Chen *et al.*^31^.

### Morphing

During thermal treatment, besides the smoothing, the surface tension also causes the microstructures to morph into a shape that minimizes the surface area^29^. Following from the conclusions drawn by Drs *et al.*^29^, the initially square-shaped suspended waveguide is expected to morph into a circular one. In fact, by measuring the dimensions of the waveguide before and after thermal annealing, it was verified that the cross-sectional area stayed constant during thermal treatment which further validates this hypothesis.

Likewise, the shape of the microresonator is also modified. From Fig. 2c, d, the straight sidewalls become round-beveled with a straight segment in-between. This is contrary to what has been reported so far^32^, where the microdisk would shrink and collapse into a microtoroid after CO_2_ laser polishing. The reason why those effects are not observed here has to do with the dimensions of the microresonator. Regardless of the microdisk’s diameter, by establishing the ratio between the radii of the pedestal to the microdisk to be 90%, the microdisk is impeded from slithering during thermal annealing and, consequently, shrinking is avoided. At the same time, this ratio is enough to stop the pedestal from deforming the WGM mode. Similarly, the height of the microdisk also influences the morphing effect. The microdisk collapses into a microtoroid akin to the work developed by Song *et al.*^32^ for heights lower than 20 µm, whereas, by increasing the height, only the bevel of its top and bottom surfaces is observed as shown in Fig. 2d.

### Excitation of WGMs

Several requirements, regarding the dimensions and alignment between the suspended waveguide and the microdisk, must be met to successfully excite WGMs in the resonator^33,34^. From numerical simulations^33^, it is concluded that the diameter of the suspended waveguide should be of a few micrometers to enhance the evanescent field and to promote optical coupling. Although these dimensions can be easily obtained with a tapered fiber fabricated through the heat-and-pull method, they are more difficult to be obtained by laser machining processes. For instance, Cheng *et al.*^31^ could not fabricate suspended waveguides in Foturan glass with diameters lower than 20 µm, whereas Ceccarelli *et al.*^20^ had to first ablate the pristine material surrounding a 20 × 90 µm^2^ Corning EAGLE X slab and then fabricate the waveguide through fs-laser writing. Here, those limitations are circumvented, and much smaller suspended waveguides, with diameters around 2–3 µm, were fabricated. Although the fabrication technique does not impose any limit to the diameter of the suspended waveguide, it was observed that smaller waveguides would break more easily during the etching process. Further, light is coupled into and out of the suspended waveguide through a lead-in and lead-out waveguide, respectively, which are fabricated following the procedure outlined in the Materials and Methods section. Given that the waveguides are geometrically aligned, the coupling losses are mainly due to mode mismatch between the lead-in (or lead-out) waveguide and the suspended waveguide. The suspended waveguide should be as small as possible to enhance the evanescent field that interacts with the microdisk. This, however, leads to an increase in the coupling losses between the lead-in (or lead-out) waveguide and the suspended waveguide. In this work, the fabrication of smaller suspended waveguides was favored at the expense of higher coupling losses. Still, it should be noted that the mode profile of the suspended waveguide is inherently related to the refractive index of the surrounding fluid, which determines the coupling losses to vary whenever the external refractive index changes.

The second criterion is related to the coupling between the suspended waveguide and the microdisk. The alignment along the vertical direction is easier to accomplish, and consists of designing the suspended waveguide centered to the microdisk. Given that chemical etching produces an almost 1:1 replica of the design and that thermal annealing barely changes the dimensions and position of both structures, this condition prevails throughout all fabrication steps.

**Figure 3:**
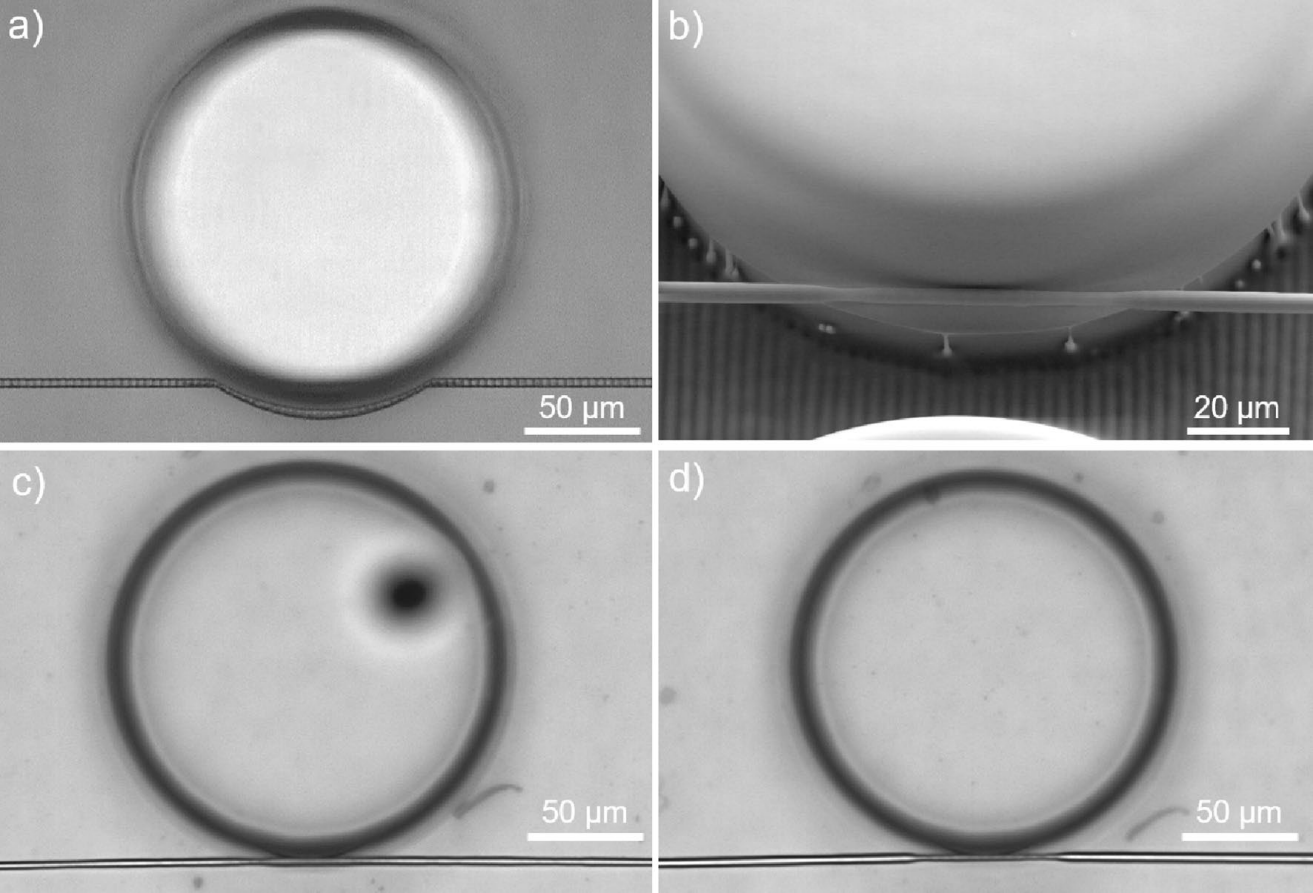
Writing technique of the suspended waveguide and morphing effect. (**a**) Top view image of the device before thermal annealing depicting the writing technique of the suspended waveguide. (**b**) SEM image of the device after annealing, where due to the morphing effect, the suspended waveguide is tangent to the microresonator. (**c**, **d**) Top view images of the device after thermal annealing, with a straight and tapered waveguide, respectively.

The biggest challenge is aligning both structures along the horizontal plane, where ideally the suspended waveguide should be nearly tangent to the microresonator^33^. As previously mentioned, the finite etching selectivity prevents this from occurring, which leads to the appearance of a micrometer-long gap between both structures that inhibits the excitation of WGMs. By adapting the geometry of the suspended waveguide and by resorting to the morphing effect, this issue can be solved as demonstrated in Fig. 3. As illustrated in Fig. 3a, the design of the suspended waveguide now consists of two symmetric straight sections, connected by an arc segment that surrounds part of the microresonator and whose curvature radius matches the microdisk’s radius. The start and end points of the arc coincide with the intersection points of the straight segments with the microresonator; although both structures distance themselves from one another during etching, their shape is preserved. During thermal annealing, the suspended waveguide straightens and gets closer to the microdisk, as seen in Fig. 3b–d. The alterations made to the design and to the fabrication procedure, namely laser-direct writing of the arc segment and thermally-induced morphing, can be used to overcome the limitations derived by the finite etching selectivity and, in turn, place the suspended waveguide tangent to the resonator. Further, the chemical etching provides a micrometric control over the gap between both microstructures, which can be used to achieve critical coupling. In addition, if the straight and arch segments are designed to have the same dimensions then, after thermal annealing, the suspended waveguide is uniform throughout its entire length, as shown in Fig. 3c. However, if in the design of the device, the dimensions of the arched section are lower than those of the straighter segments, the suspended waveguide becomes tapered after thermal annealing, as demonstrated in Fig. 3b, d. This way, it is possible to further narrow the suspended waveguide and, in turn, enhance the evanescent field without compromising the mechanical stability of the suspended waveguide.

**Figure 4:**
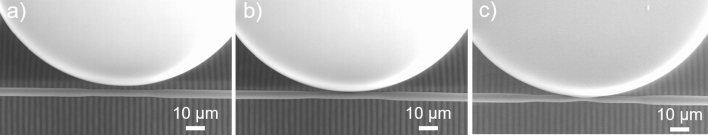
Coupling between the Suspended Waveguide and the Microdisk. SEM images illustrating the possible assembly scenarios: the suspended waveguide is (**a**) separated from the microdisk, (**b**) tangent to the microdisk, and (**c**) merged with the microdisk.

Three different situations, shown in Fig. 4a–c, illustrate the control that can be obtained depending on when the etching reaction is stopped. In particular, if it is stopped when the straight segment of the suspended waveguide is tangent to the microdisk then, after thermal annealing, the arched section will straighten and be tangent to the microresonator, as shown in Fig. 3b–d and in Fig. 4b. This is different from what was reported by Song *et al.*^32^, where a tapered fiber would deform after being welded to the microtoroid, thereby placing the device in the over-coupling regime. If the reaction is stopped later both structures become distanced, whereas if it is stopped earlier the suspended waveguide merges with the microdisk and becomes deformed, as shown in Fig. 4a, c, respectively.

Figures 3b and 4a–c also reveal that the bottom surface of the microfluidic channel has a corrugated profile and that the microdisk’s pedestal presents some defects. These profiles are a consequence of the finite etching selectivity, and aggravated by the fact that the duration of the etching reaction is determined by the final position of the suspended waveguide relative to the microdisk. As a result, the layers beneath the microdisk are etched for a shorter time period, in comparison to the rest of the material, which translates into a poorer surface quality. Still, these irregularities do not introduce optical losses to the device, and can easily be solved by using a closely spaced hatching scans in the layers defining the bottom of the microfluidic channel.

**Figure 5:**
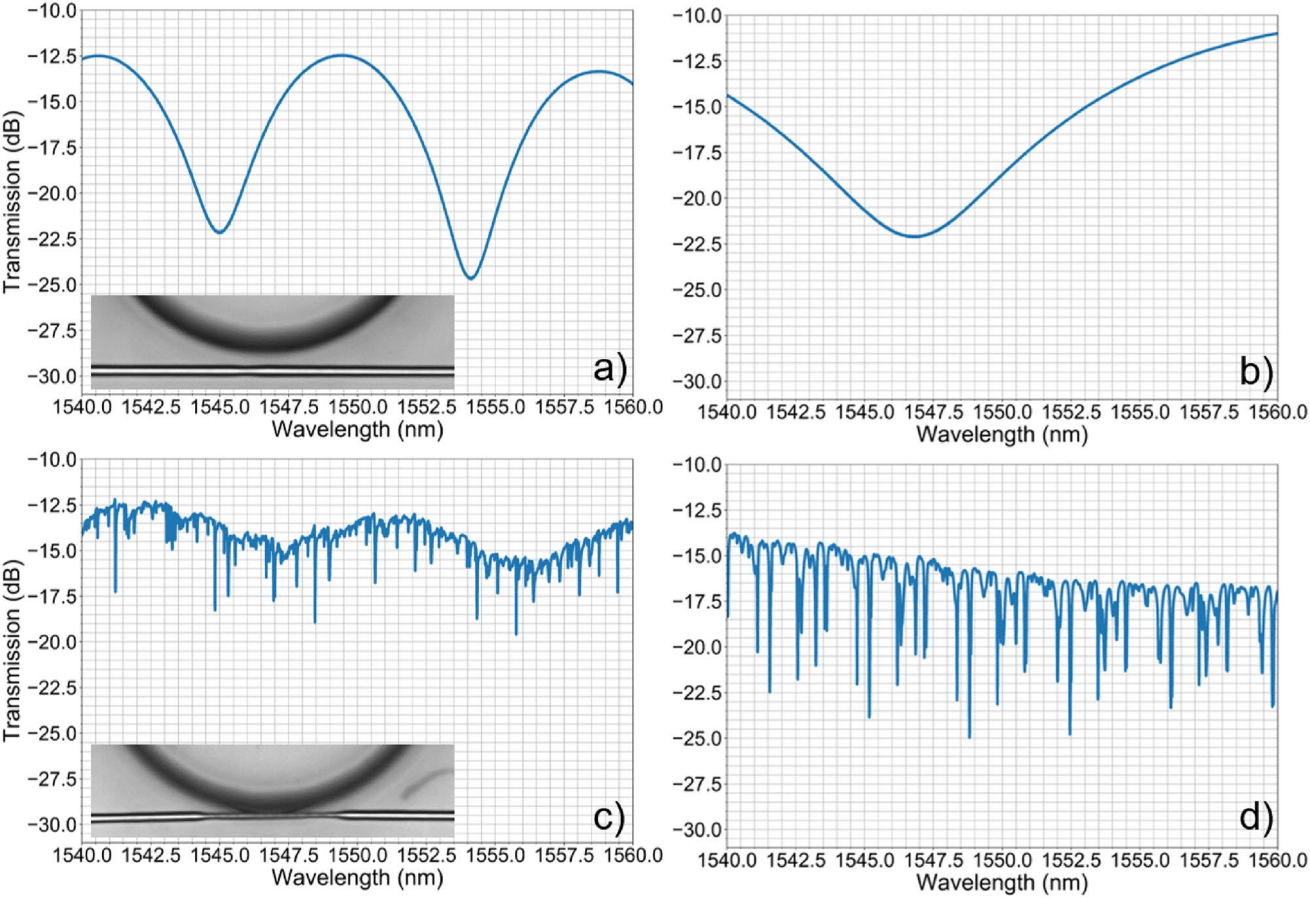
Transmission spectrum of the suspended waveguide. (**a**, **b**) Spectrum in air and deionized water, respectively, of a suspended waveguide with diameter of 3.7 µm, distanced of 2.2 µm from a 72 µm radius microdisk. (**c**, **d**) Spectrum in air and deionized water, respectively, of a suspended waveguide with waist diameter of 2.4 µm and tangent to a 72 µm radius microdisk. The inset images show a top view image of the respective device. The measurements were made from high magnification SEM images.

In the situation displayed in Fig. 4a, the suspended waveguide is 2.2 µm away from the microdisk border. Accordingly, no WGMs are excited as can be confirmed from the transmitted spectrum of Fig. 5a, b in air and deionized water, respectively. Instead, a periodic modulation is observed, which is caused by Mach–Zehnder interference (MZI) between the fundamental mode of the suspended waveguide and uncoupled light that propagates across the microfluidic channel^35^. Kelemen *et al.*^25^ reported this same issue, but were able to avoid it by writing a bended suspended waveguide with the input and output transversely distanced.

The scenario displayed in Fig. 4b enables the excitation of WGMs as evidenced from the transmitted spectra shown in Fig. 5c, d, where a suspended waveguide with waist diameter of 2.4 µm is put in contact with a microdisk with radius and height of 72 µm by 50 µm, respectively. Multiple modes of the resonator are excited, due to the measurements being made with linearly polarized light at an unknown angle that causes both TE (transverse electric) and TM (transverse magnetic) modes to be excited, and due to the large dimensions of the microdisk which supports multimode propagation^36^. Still, a dominant resonance periodically spaced by 3.6 nm is seen in both graphs. The measured free spectral range is in good agreement with the expected value, whereas the apparent independence from the external media is associated to a weak variation of the effective index of the excited WGM with the surrounding refractive index. Ideally, to reach the critical coupling condition, the suspended waveguide should be distanced by 200–400 nm from the microdisk^37^. This could be achieved if the etching reaction was stopped slightly earlier as explained above. Further, this requirement can be reached more easily if an etching agent with higher selectivity or which attacks the pristine medium at a slower rate is used instead of HF acid at a volumetric concentration of 10%. Also, in spite of the WGM spectrum overlapping with the MZI spectrum defined earlier, the period of the MZI modulation is an order of magnitude higher than the free spectral range of the WGM spectrum, for all surrounding fluids tested. Ergo, the presence of the overlaying modulation does not interfere with the optical characterization of the whispering-gallery resonator.

The condition illustrated in Fig. 4c places the device in the over-coupling regime, which is accompanied by an increase in the insertion losses. Song *et al.*^32^ and Kelemen *et al.*^25^ also observed this effect, and added that the Q-factor decreases in this regime.

### Optical characterization

To demonstrate its applicability as a refractive index sensor, the response of the device (shown in Fig. 3d and in the inset of Fig. 5c) against the surrounding media was characterized. Different Cargille fluids (series AA), with refractive index spanning from 1.296 to 1.363 at 1550 nm, were successively inserted in the microfluidic channel and the transmission spectrum was measured. The device was thoroughly cleaned in-between measurements, and it was confirmed that no contaminants were inside the microfluidic channel nor were they deposited in the surface of either microstructures. Further, the transmission spectrum of an empty microfluidic channel was measured prior to filling the microfluidic channel with testing fluids, having always obtained a spectrum equal to the reference signal shown in Fig. 5c. This indicates that the device is clean and that the results are repeatable. To simplify the following analysis, the measurements were made with the input beam linearly polarized to excite only the TM modes of the microresonator.

**Figure 6:**
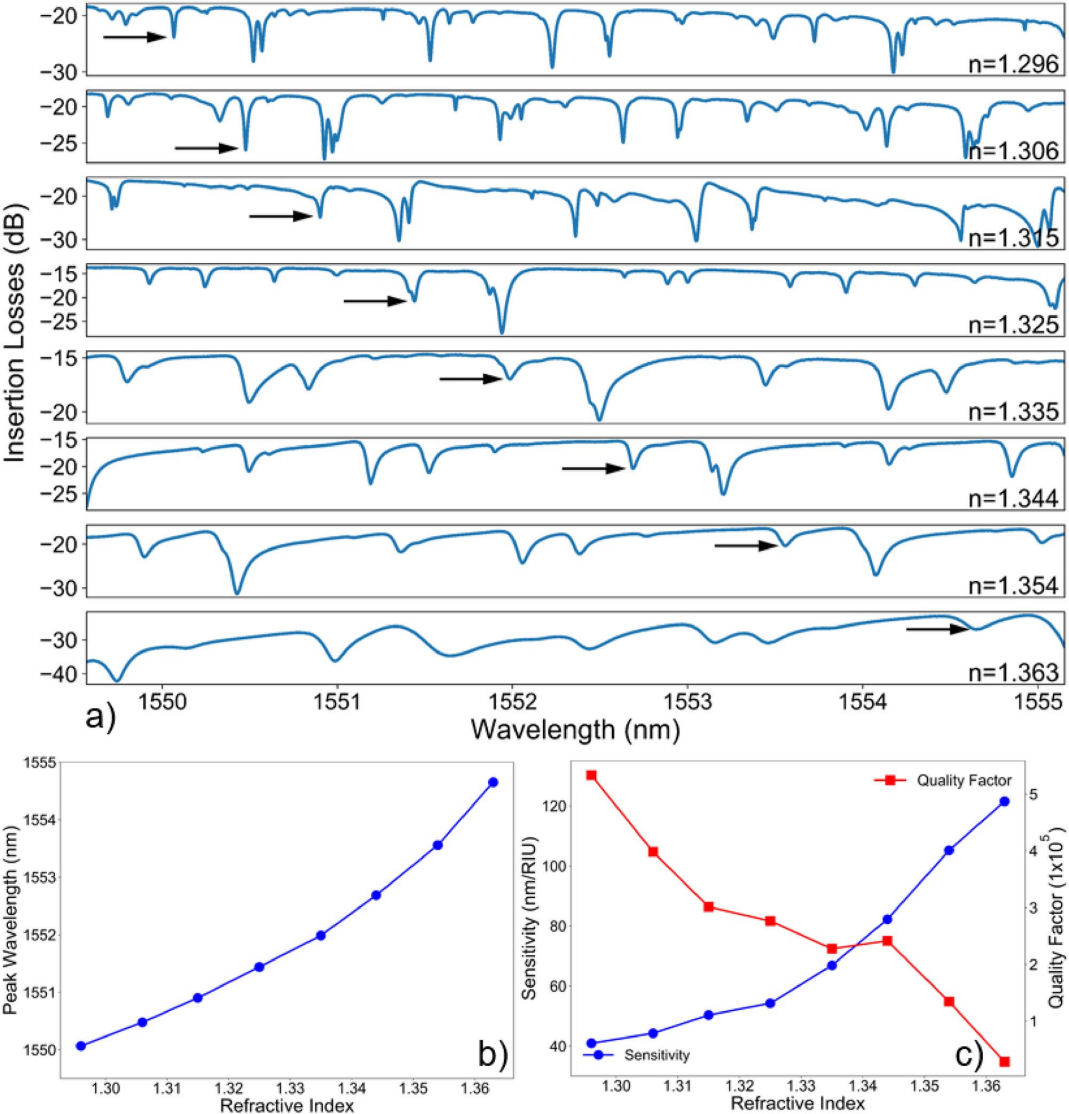
Optical characterization against refractive index. (**a**) Transmission spectrum of the suspended waveguide, described in Fig. 5c–d, against different fluids surrounding the microresonator. The arrow signals the WGM mode under analysis. (**b**) Evolution of the resonant wavelength with the external refractive index. (**c**) Sensitivity (circular markings) and Q-factor (square markings) against the surrounding media.

Overall, the resonances broaden and weaken as the refractive index increases, which is accompanied by a reduction in the number of excited modes. These results are summarized in Fig. 6, where it was tracked the behavior of the resonance located at 1550 nm. As the external refractive index increases, the resonance shifts non-linearly to higher wavelengths. According to Eq. (1), which defines the resonant wavelength (${\lambda }_{res}$) as a function of the radius of the microdisk ($R$) and of the effective index of the propagating WGM (${n}_{eff}$) with azimuthal number $m$, these results also indicate that the effective refractive index of the WGM is increasing.

1 $${\lambda }_{res}=\frac{2\pi R{n}_{eff}}{m}$$

Luo *et al..* reached a similar conclusion after simulating the behaviour of the effective refractive index of optical modes of a microdisk resonator as the refractive index contrast between the host and surrounding media decreased^38^. The sensitivity to refractive index variations of the surrounding medium, displayed in Fig. 6c, is obtained by plotting the first derivative of the curve shown in Fig. 6a. The behavior shown by the sensitivity curve, where a higher sensitivity is obtained at higher refractive indices, is expected given that the effective refractive index of the WGM under analysis shifts non-linearly with the surrounding medium. A maximum sensitivity of 121.5 nm/RIU is obtained at an index of 1.363, with a detection limit^39^ of 7.0 × 10^–4^. For refractive indices near that of aqueous solutions, the sensitivity is 40 nm/RIU. For comparison, Song *et al.*^32^ and Kelemen *et al.*^25^ obtained a linear sensitivity of 61 ± 1 nm/RIU between 1.3344 and 1.3840, and of 220 nm/RIU around the refractive index of water, with a 25 µm radius polymeric microring and with a 40 µm fused silica microtoroid, respectively.

After fitting the resonance with a Lorentzian function, the peak width (full-width at half-maximum) and the Q-factor were computed^39^. Figure 6c shows that the Q-factor decreases from 5.33 × 10^5^ to 0.28 × 10^5^ as the refractive index increases from 1.296 to 1.363, which is attributed to weaker light confinement inside the microresonator^40^. Still, the measured Q-factor is on par with what was reported by Song *et al.*^32^, and is two orders of magnitude higher than what was reported by Kelemen *et al.*^25^.

## Discussion

In this work, it was constructed a label-free optofluidic sensor entirely made of fused silica, which integrates a whispering-gallery mode resonator, being excited by the evanescent field of a suspended waveguide, inside a microfluidic channel. The device is fabricated by fs-laser micromachining followed by chemical etching and thermal annealing. The introduction of this procedure decreases the surface roughness of both microstructures to tens of nanometers, thereby improving the quality factor. Further, thermal annealing together with the design of a bended suspended waveguide surrounding the microdisk enables the accurate positioning of the waveguide tangent to the microresonator. The versatility provided by fs-laser micromachining also enables us to accurately control the dimension and geometry of both microstructures in order to attain optimal phase-matching. Specifically, the control over the dimensions of the arc segment within the coupling region can be used to enhance the spatial overlap between the evanescent field of both modes and to improve optical coupling. The morphing tool described here solves numerous practical issues regarding the handling and the applicability of the device. The fabrication and experimental results are repeatable with no surface fouling being observed with the test fluids used on these experiments, while the monolithic construction guarantees that the suspended waveguide and resonator are robust and aligned at all times. Other results associated with surface fouling can, for example, be observed with biological samples, but that was not assessed during this work. The device shows a sensitivity of 40 nm/RIU and a quality factor of 2 × 10^5^ for refractive indices near that of aqueous solutions, akin to what has been reported for similar devices and enough for most biosensing applications. Fluid handling capabilities can still be incorporated within the microfluidic channel^18, 24^, whereas Kelemen *et al.*^25^ demonstrated these optical systems can also be used in biosensing applications.

Still, improvements to the current device can be made. A higher control of the distance between the suspended waveguide and the microresonator can be achieved by exploiting etchants with higher etching selectivity. Further, optimal phase-matching and operation in the critical coupling regime can be achieved by relying on numerical simulations to determine the most suitable dimensions for the structures as well as the gap between them. However, the measurement of the real gap can be hindered by experimental issues, such as charge effects during SEM analysis. Different geometries can also be explored to fully remove the influence of the Mach–Zehnder interference on the whispering-gallery mode spectrum. In particular, a possible solution is to fabricate two suspended waveguides, placed tangent to and on opposite sides of the resonator. In this geometry, light propagating forward across one of the waveguides excites WGMs of the microresonator, which are themselves coupled to the other waveguide where light propagates backwards and whose spectrum is measured. Meanwhile, the microfluidic channel can also be fabricated beneath the surface of the fused silica substrate or sealed with a PDMS layer where, by opening two holes to the cover layer, fluids can be drawn into and out of the channel. In that context, it is also important to determine the limits of operation of the device by measuring the maximum flow rate supported by the microfluidic channel before causing breakage of the microstructures.

## Materials and methods

### Fabrication of the device

The device is fabricated in a pure fused silica substrate (Suprasil 1), with dimensions of 25 × 25 × 1 mm^3^. First, the side facets of the substrate are polished in order to minimize the optical coupling losses to the lead-in and lead-out waveguides. The microfluidic channel is then fabricated in a laser direct writing workstation (Workshop of Photonics) by irradiating the substrate with the second harmonic of a fiber amplified fs-laser system (Amplitude Systèmes HP). The laser beam, operating at 515 nm with an approximate pulse duration of 250 fs and repetition rate of 500 kHz, is focused inside the substrate by a 0.42 numerical aperture plan achromat objective (Mitutoyo M Plan Apo NIR 50 ×). Aerotech direct-drive stages (ANT130XY-110 PLUS and ANT130V-5 PLUS) are responsible for scanning the substrate in relation to the laser beam, according to the user-defined path. The three-dimensional design of the microfluidic channel is sliced in several horizontal layers, vertically spaced by 5 µm, which are written sequentially from the bottom to the top. The properties (position, shape, and size) of the suspended waveguide and microdisk are stored in these layers, which are further hatched into parallel tracks, regularly spaced by 3 µm, which define the path of the laser beam. Each of these lines is written with a scanning speed of 3000 µm/s and pulse energy of 60 nJ. Additionally, the lines are oriented along the minor axis of the microfluidic channel in the layers containing the suspended waveguide, and oriented along the major axis in the remaining layers. Further, these tracks end 1 µm from the contour of the suspended waveguide and of the microdisk, whose corresponding scans are written with a translation velocity of 500 µm/s and pulse energy of 50 nJ. With the exception of the contour of the microdisk and its pedestal, the polarization of the laser beam is orthogonal to the scanning direction. When designing the device, it is important to bear in mind that the dimensions of the structures, after laser direct writing, have to be corrected by the width and height of a single modification track, which are 2 µm and 10 µm at the previous scanning conditions, respectively. The laser direct writing process takes less than 30 min, after which the substrate is immersed for 50 min in an ultrasonic bath (Branson 2510) of a 10% hydrofluoric (HF) acid solution at 30 °C, which will remove preferentially the regions irradiated by the fs-laser beam. Thermal annealing is then performed in a closed furnace (Carbolite RHF-1500). The temperature of the substrate is ramped up to the setpoint value at an initial rate of 15 °C/minute, which starts decreasing to less than 1 °C/minute as the temperature surpasses 1000 °C. It takes around five hours for the system to reach the target temperature, after which the substrate is held at this temperature for an additional five hours, before cooling down slowly to room temperature. Thermal annealing is conducted at atmospheric pressure without atmosphere control. Lastly, the lead-in and lead-out waveguides are written through fs-laser direct writing, using the same workstation mentioned above.

To minimize coupling losses due to geometrical mismatches between the lead-in (or lead-out) waveguide and the suspended waveguide, the substrate is aligned so that all waveguides are parallel to each other and their respective optical axes coincide. Both axes are aligned along the horizontal and vertical directions within a 0.1 µm and 1 µm error, respectively. The waveguides terminate 20 µm from the border of the microfluidic channel, and are written with a scanning speed of 200 µm/s, pulse energy of 100 nJ and with the laser beam polarized along the scanning direction.

### Optical characterization

To measure the transmission spectrum of the device, a continuous swept laser source was coupled into a single-mode fiber (SMF-28) and butt-coupled to the entrance of the lead-in waveguide. Light coming from the lead-out waveguide is coupled into another optical fiber, and collected by a spectral analyzer (FS22SA Spectral Analyzer Industrial BraggMETER) operating from 1500 to 1600 nm with a 1.0 pm resolution. The substrate is placed on an Elliot Martock MDE881 stage with piezo controls (Dali E-2100) and special holders for precise alignment of the input/output fibers with the lead-in/lead-out waveguides, respectively. Index matching (Cargille series AA ${n}_{D}^{25^\circ{\rm C} }=1.4580\pm 0.0002)$ was used to minimize Fresnel reflections at the input/output facet. The spectra reported in Fig. 5 were obtained with light linearly polarized along an unknown direction, whereas the ones displayed in Fig. 6 were obtained with a light beam linearly polarized parallel to the surface of the substrate. This was achieved by placing, in-between the optical source and the substrate, an in-line fiber polarizer (ThorLabs ILP1550 PM-APC) followed by a polarization-maintaining fiber (Fujikura PM1550), which is assembled on a rotary fiber holder (Elliot Martock) that controls the input polarization angle. All measurements were made at 20 ± 1 °C.

### Profilometer

The surface profile of a microfluidic channel, with surface area of 800 × 500 µm^2^, was measured through contact profilometry (Dektak Bruker profilometer) with a tip stylus terminating in a 45° cone and end radius of 2.5 µm. To compute the root-mean squared roughness, a band-pass filter^41^ was applied to the measured surface profile in order to remove the influence of the waviness and nominal profiles (low-frequency component) and the noise profile associated to the measurement resolution (high-frequency component). The cut-off frequencies were chosen in a way that minimizes the skewness of the resulting surface.

### Microscopy

Optical microscopy and scanning electron microscopy (SEM) were both used to characterize the devices. A Leica microscope unit was used to observe, with 200 × ampliation, the top and cross-section profiles of the microstructures. For SEM imaging, the samples were first coated with an Au/Pd thin film by sputtering, using the SPI module Sputter Coater equipment. A high resolution (Schottky) Environmental Scanning Electron Microscope with X-Ray Microanalysis and Electron Backscattered Diffraction analysis (FEI Quanta 400 FEG SEM / EDAX Genesis X4M), operating at low vacuum, was then used to observe the microstructures.
